# LLDPE Composites with Nanosized Copper and Copper Oxides for Water Disinfection

**DOI:** 10.3390/polym12081713

**Published:** 2020-07-30

**Authors:** Yanna Gurianov, Faina Nakonechny, Yael Albo, Marina Nisnevitch

**Affiliations:** Department of Chemical Engineering, Ariel University, Kyriat-ha-Mada, Ariel 4070000, Israel; yannag@ariel.ac.il (Y.G.); fainan@ariel.ac.il (F.N.); yaelyt@ariel.ac.il (Y.A.)

**Keywords:** copper nanoparticles, cuprous oxide nanoparticles, cupric oxide nanoparticles, linear low-density polyethylene, antibacterial surfaces, antibacterial activity

## Abstract

Consumption of contaminated water may lead to dangerous and even fatal water-borne diseases. Disinfection of drinking water is the most effective solution for this problem. The most common water treatment methods are based on the use of toxic disinfectants. Composites of polymers with nanosized metals and their oxides may become a good alternative to the existing methods. Expanding the scope of our previous publication, copper, cuprous, and copper oxide nanoparticles were immobilized onto linear low-density polyethylene by a simple thermal adhesion method. The antibacterial efficiency of the immobilized nanoparticles was tested against Gram-negative *Escherichia coli* and Gram-positive *Staphylococcus aureus* in batch experiments and for the first time the efficiency of these composites is reported for continuous flow regime. Immobilized copper and cuprous oxide nanoparticles demonstrated a high ability to eradicate bacteria after 30 min. These composites showed no or very limited leaching of copper ions into the aqueous phase both in the presence and in the absence of a bacterial suspension. Immobilized copper and cuprous oxide nanoparticles can be used for batch or continuous disinfection of water.

## 1. Introduction

According to data of the World Health Organization (WHO), almost two billion people worldwide use contaminated drinking water. This leads to the spread of dangerous and even fatal water-borne diseases such as diarrhea, cholera, dysentery, typhoid, and poliomyelitis [1]. Spread of these diseases via drinking water can be prevented by disinfection [2]. The most commonly used and cheapest disinfectant is chlorine, which deactivates microorganisms in drinking water treatment plants [3]. This process has serious drawbacks due to generation of toxic and carcinogenic products such as trihalomethanes and haloacetic acids [4,5]. Another chemical disinfectant is ozone [6]. However, ozonation can lead to the formation of a toxic bromate by reacting with bromide present in water [7]. Non-chemical disinfectants, such as UV radiation, are powerful against protozoa, bacteria, fungi, and viruses. Use of this method is limited, since turbidity of a water source decreases UV transmittance to the microorganisms [8]. Modern and more promising alternative water treating technologies can be based on the use of polymers coated with nanoparticles (NPs). Nanotechnology is widely applied in biomedicine, food technology and waste-water treatment [9,10]. Coating polymers with inorganic materials such as metal or metal oxide NPs, or embedding the latter into polymers, can improve the mechanical and chemical properties of polymeric matrices [11]. These polymer-based nanocomposites can serve as antibacterial, bacteriostatic, antifouling, and self-cleaning surfaces [12,13,14]. Copper and copper oxide NPs are very potent in water disinfection applications [15], since they exhibit high antibacterial, antifungal and antiviral properties [14,16,17,18]. Previous studies showed that the antibacterial activity of nanosized Cu, Cu_2_O, and CuO depends on the morphology and size of these NPs, as well as on the dissolution ability of copper species in the surrounding media [19]. The exact mechanism of the antimicrobial action of nanosized copper-based particles is still not totally clear, but it is known that copper NPs penetrate into the outer membrane of microbial cells better and faster than copper microparticles. When passing through the cell membrane, NPs undergo ionization and the chain reaction of contact killing is initiated [20]. Copper ions participate in a Fenton-like reaction with respiratory byproducts of aerobic microbes, such as hydrogen peroxide $H_{2}O_{2}$ (1) and superoxide ${O_{2}}^{-}$ (2) [20,21].

(1) $$Cu^{+}+H_{2}O_{2}\to Cu^{2+}+OH^{-}+OH^{.}$$

(2) $$Cu^{2+}+{O_{2}}^{-}\to Cu^{+}+O_{2}$$

The hydroxyl radical generated in the Fenton-like reaction (1) can lead to peroxidation of proteins, lipids, and DNA [22,23]. Another possible mechanism of copper ion action against microorganisms is inactivation of important enzymes by binding to sulfhydryl groups of cysteine amino-acidic residues of proteins and changing their structure. In addition, formation of reactive oxygen species (ROS), causing membrane damage and leading to cell death, takes place, [24]. Researchers currently believe that copper and its oxides differ in their antibacterial mechanisms from copper ions. Applerot et al. suggested that the antibacterial activity of nanocrystalline CuO can be attributed to production of ROS (mostly anionic superoxide) on the surface of the NPs and not to a release of soluble copper ions [25]. Meghana et al. assumed that the antibacterial activity of cuprous oxide NPs (Cu_2_ONPs) against *E. coli* can be related to interaction of NPs with intracellular proteins such as fumarase A, an iron cluster containing enzyme, and not to a reaction of NPs with ROS [26]. We have previously shown that Cu_2_ONPs attached onto polyethylene by thermic adhesion have good activity against Gram-positive and Gram-negative bacteria [27]. The aim of the present study was to develop a continuous water disinfection process using NPs of copper and copper oxides immobilized onto polymeric surfaces. 

## 2. Materials and Methods 

### 2.1. Materials

Linear low-density polyethylene (LLDPE) was purchased from Sigma-Aldrich^®^ (Saint Louis, MO, USA). Cu_2_ONPs with a size of 18 nm, CuNPS and CuONP with a size of 40 nm were purchased from US Research Materials (Houston, TX, USA). 

### 2.2. Thermal Adhesion of CuNPs, Cu_2_ONPs, and CuONPs onto the LLDPE Polymer

A strip of Kapton polyimide film 0.127 mm thick (Shagal Marketing Solutions Ltd., Modiin, Israel) was placed on the lower plate of heat press machine (Dulytek^®^ DM1005, Dulytek, Seattle, WA, USA), on which 1 g of LLDPE pellets were evenly distributed. A second strip of Kapton polyimide film was placed above the pellets to avoid a direct contact between the polymer and the plates of the press machine. The polymer was melted at 125 °C using the maximal pressure of the machine under 450 $kg_{f}$ for 3 min. The upper polyimide film was then removed from the sample and 0.15 g of NPs were dispersed on the molten polymer using a sieve. The NPs and the molten polymer were covered with another polyimide film and the upper plate was slightly pressed in for a few seconds. The samples were cooled to a room temperature, and both strips of the Kapton polyimide film were gently removed. The thickness of the samples was measured with a digital 150 mm caliper (Roher-tools, Roher^®^, Ramla, Israel).

### 2.3. Bacterial Growth

Cultures of Gram-positive *S. aureus* (ATCC 11522) and Gram-negative *E. coli* (ATCC 9723e) were grown in brain heart infusion agar (BH, Acumedia, Lansing, MI, USA) and Luria Bertani agar (LB, Himedia^®^, Mumbai, India), respectively, for 24 h, after which the inoculum was transferred into a corresponding broth medium and grown at 37 ± 1 °C and shaking at 150 rpm until reaching OD_660nm_ (optical density) = 0.3. The bacterial suspensions were diluted with sterile saline to a final concentration of 10^2^ or 10^3^ cells·mL^−1^. 

### 2.4. Antibacterial Activity Assay 

The antibacterial activity of samples of free and coated LLDPE with CuNPs, Cu_2_ONPs and CuONPs was tested in a batch regime as follows: 20 mL of bacterial suspension at a concentration of 10^3^ cells·mL^−1^ in sterile saline were placed into a 250 mL sterile Erlenmeyer flask with 0.15 g of NPs powder or 1 g of NPs-LLDPE composites containing 0.15 g of NPs and incubated at 25 ± 1 °C with shaking at 120 rpm for 30 min. The samples were then diluted in duplicates by one decimal dilution and 100 µL of these samples were distributed onto BH or LB agar plates in the case of *S. aureus* and *E. coli*, respectively. The plates were incubated overnight at 37 ± 1 °C and the bacterial colony forming units (CFU) were counted using a colony counter Scan 500 (Interscience, Saint-Nom-la-Bretèche, France). 

The antibacterial activity of samples of LLDPE coated with CuNPs and Cu_2_ONPs was tested in a continuous regime as follows: three samples of 1 g of LLDPE-NPs were rolled and inserted into horizontal 1 × 25 cm columns, after which the bacterial suspension in saline was allowed to flow through the columns at flowrates of 0.144–0.166 mL/min using a multi-channel peristaltic pump (Ismatech ISM1089C Ecoline, Cole-Parmer GmbH, Futtererstr. Wertheim, Germany) and tubes with an inner diameter of 0.95 mm (Tygon^®^ E-Lab, Cole-Parmer Scientific Experts, IL, USA). The *S. aureus* suspension was used at a concentration of 10^3^ cells·mL^−1^ and the *E. coli* suspension was used at a concentration of 10^2^ cells·mL^−1^. The control column contained a bacterial suspension only, flowing through the column at the same flowrates. Samples from the inlet and the outlet of the columns were taken after 7 h for *S. aureus* and 20 h for *E. coli* and tested for the bacterial concentration by the live cell count method as described above. During the first week of experiments, the source suspension of bacteria was replaced daily by a fresh source, after which the source suspension was replaced by a fresh one only twice a week.

### 2.5. Testing Copper Ion Leakage from Immobilized NPs into a Saline Solution and Bacterial Suspensions 

Leaching of copper ions from immobilized NPs was tested either in saline in a batch experiment or in saline with bacterial suspensions in a continuous regime. The former examination was carried out as follows: 2 g of LLDPE with 0.15 g of immobilized NPs were added to 500 mL of saline and stirred at 120 rpm with a magnetic stirrer for one month. Samples of 1 mL were taken twice a week. The samples were diluted with 9 mL of distilled water and filtered through PVDF Millex^®^-GV membranes with a 0.22 µm pore size (Merck Millipore Ltd., Carrigtohill, Ireland). Testing copper release from immobilized CuNPs and Cu_2_ONPs in a continuous regime was performed in the samples taken for the antibacterial tests. Bacterial suspension was sampled at the inlet and outlet of the column and filtered through Millex^®^-GV membranes with a 0.22 µm pore size. The copper ion concentration in the samples was measured using the ICP-AES (Spectro Arcos, Ametek^®^, Berwyn, PA, USA) instrument.

### 2.6. SEM Analysis of Immobilized NPs

Imaging of surfaces and cross-sections of immobilized CuNPs, Cu_2_ONPs, and CuONPs was performed with a SEM microscope (Tescan MAIA3, Triglav™, Brno, Czech Republic). The samples were placed onto a carbon tape and covered with a 10-nm carbon layer using a Q150T ES Quorum coater (Quorum Technologies Ltd., Laughton, UK) under a sputter current of 12 mA for 30 s. SEM measurements were performed at operating voltages of 5 and 15 kV and at magnifications of ×650, ×1.10 k and ×60 k. The samples were detected with In-beam SE and SE detectors. Energy dispersive X-ray spectroscopy (EDS) analysis of the samples was performed in SEM mode under a resolution of 127 eV using a X-Max^N^ SDD detector 51-xmx1010 (Oxford Instruments NanoAnalysis, High Wycombe, UK).

### 2.7. XRD Analysis of the Powder and Immobilized NPs

The phase composition of the powder and immobilized CuNPs, Cu_2_ONPs, and CuO were studied by XRD analysis using a Rigaku SmartLab SE X-ray powder diffractometer with Cu Kα radiation (λ = 0.154 nm) for phase identification. Full pattern identification was made by the SmartLab Studio II software package, version 4.2.44.0 (Rigaku Corporation, Tokio, Japan). Materials identification and analysis were performed by the ICDD base PDF-2 Release 2019 (Powder Diffraction File, ver. 2.1901). XRD patterns were received at 40 kV and 40 mA. For the powder samples the diffractograms were obtained with Bragg–Brentano geometry. The XRD patterns were recorded in the 2Θ range of 20–80° with a step size 0.01° and speed of 4°/min. For immobilized NPs the grazing incidence geometry with an incident angle of 0.5° was applied. The XRD patterns were recorded in the 2Θ range of 20–80° with a step size 0.01° and speed of 0.5°/min.

### 2.8. Statistical Analysis

The results were obtained from at least three independent experiments carried out in duplicates and analyzed by single-factor ANOVA analyses. Quantitative results are presented as the mean *±* standard error.

## 3. Results and Discussion

### 3.1. Immobilization of Cu and Its Oxide NPs onto a Polymeric Surface 

Composites of LLDPE with NPs of copper and its oxides were prepared by thermal adhesion, since samples of Cu_2_O immobilized onto the polymer by this method were found to be the most active against *S. aureus* and *E. coli* cells [27]. In addition, no leaching of copper ions into tap water was registered in the batch regime [27]. In the present study, immobilization of NPs was carried out using a heat press machine. This enabled obtaining thin samples with a thickness of 0.2 to 0.5 mm. These thin composites were very convenient for rolling into a spiral and inserting them into a column for further use in continuous regime experiments.

**Figure 1:**
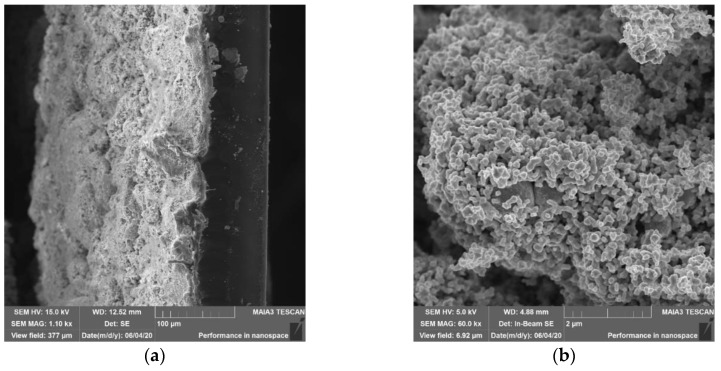

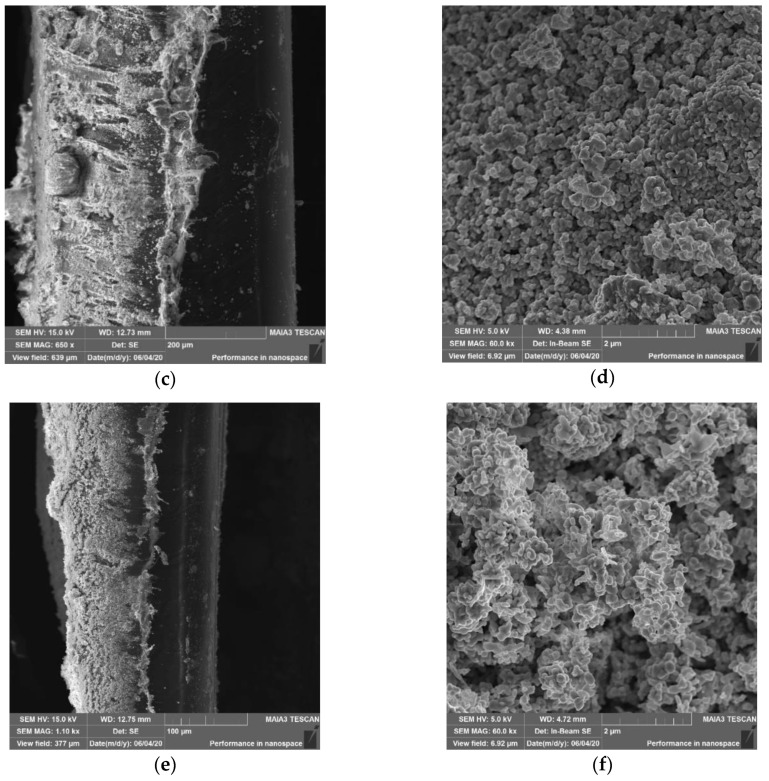
Cross-section (**a**,**c**,**e**) and surface (**b**,**d**,**f**) SEM micrographs of nanoparticles (NPs) immobilized onto linear low-density polythene (LLDPE) by thermal adhesion: CuNPs (**a**,**b**); Cu_2_ONPs (**c**,**d**); CuONP (**e**,**f**).

The obtained composites were examined by scanning electron microscopy (SEM) (Figure 1). The cross-section images (Figure 1a,c,e) present a nanoparticle coating layer on the left side of the image and the polymer itself can be seen on the right side. The micrographs show that the polymeric surfaces are completely and evenly covered with NPs (Figure 1b,d,f). The thickness of the obtained composites was 279 ± 8 µm for LLDPE/CuNPs, 541 ± 20 µm for LLDPE/Cu_2_ONPs, and 188 ± 17 µm for LLDPE/CuONPs. 

**Figure 2:**
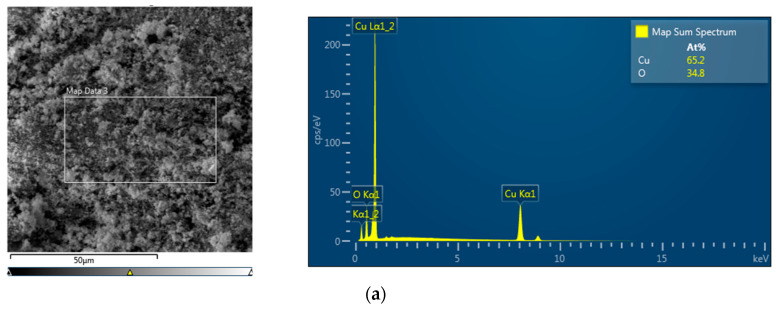

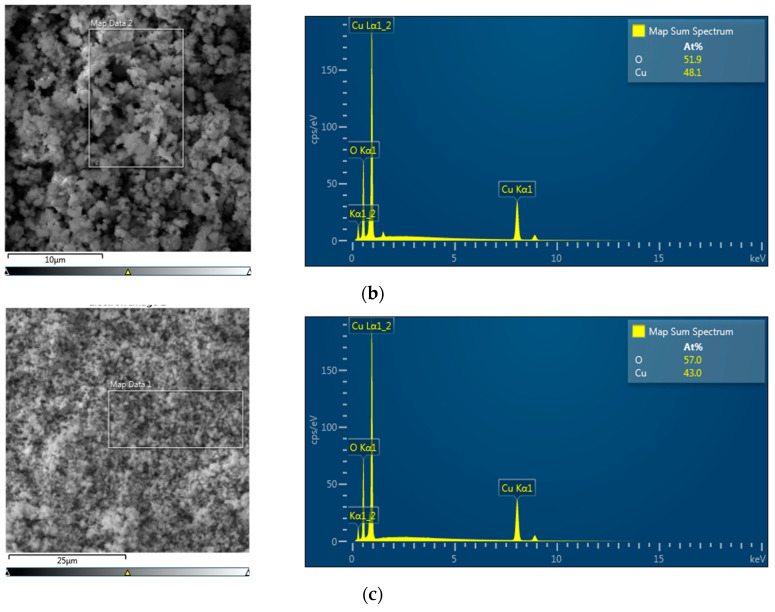
EDS images of carbon-coated NPs immobilized onto LLDPE: (**a**) CuNPs; (**b**) Cu_2_ONPs; (**c**) CuONPs.

The surfaces of the composites were analyzed by EDS. The left panels of Figure 2 show the coating of NPs on the polymeric surface. The right panels show the molar fractions of copper and oxygen atoms. For composites containing CuNPs and Cu_2_ONPs (Figure 2a,b, respectively), the atomic fraction of oxygen was higher than expected, probably due to partial oxidation of copper and cuprous oxide NPs under ambient conditions. The same phenomenon was observed in our previous work [27]. However, the copper-oxygen molar ratio in immobilized CuONPs was close to 1:1, as anticipated (Figure 2c).

**Figure 3:**
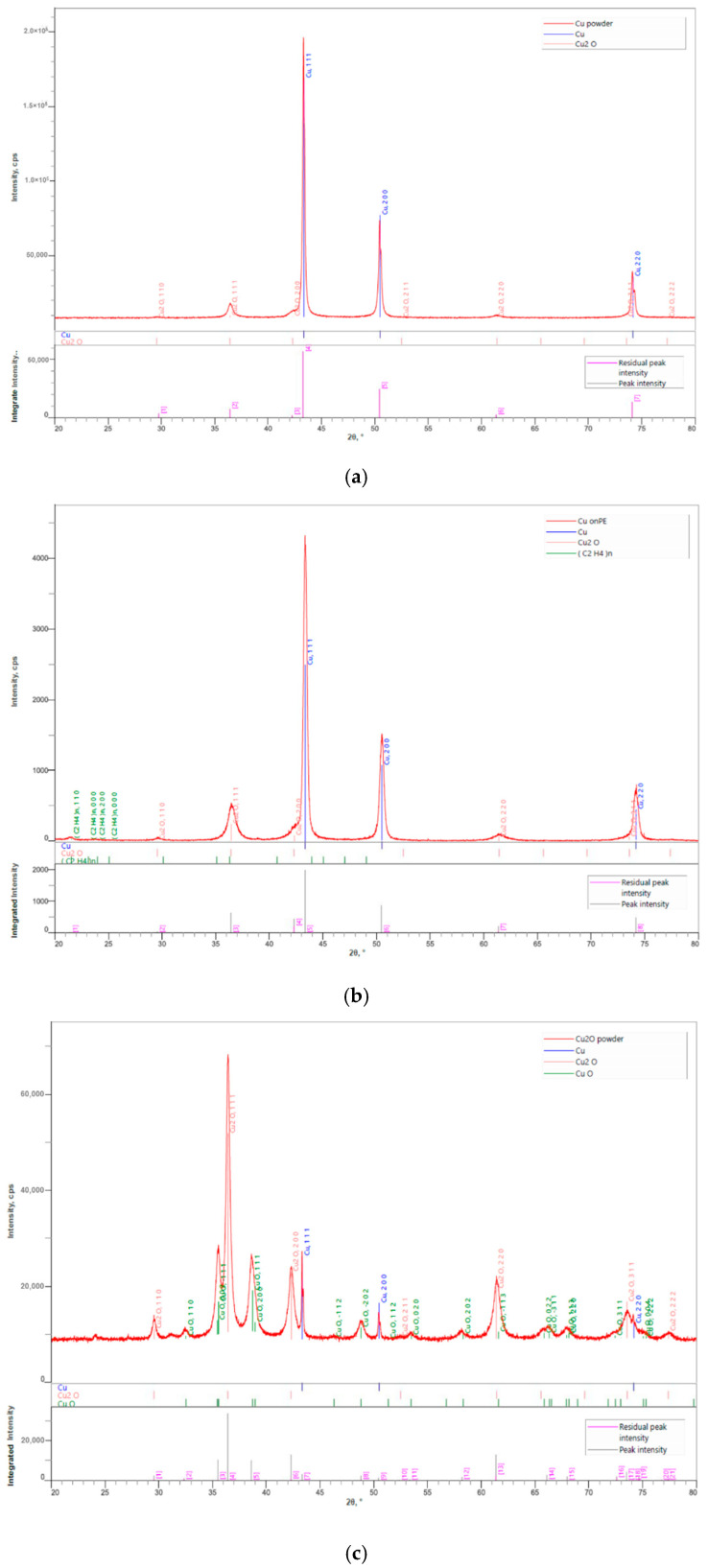

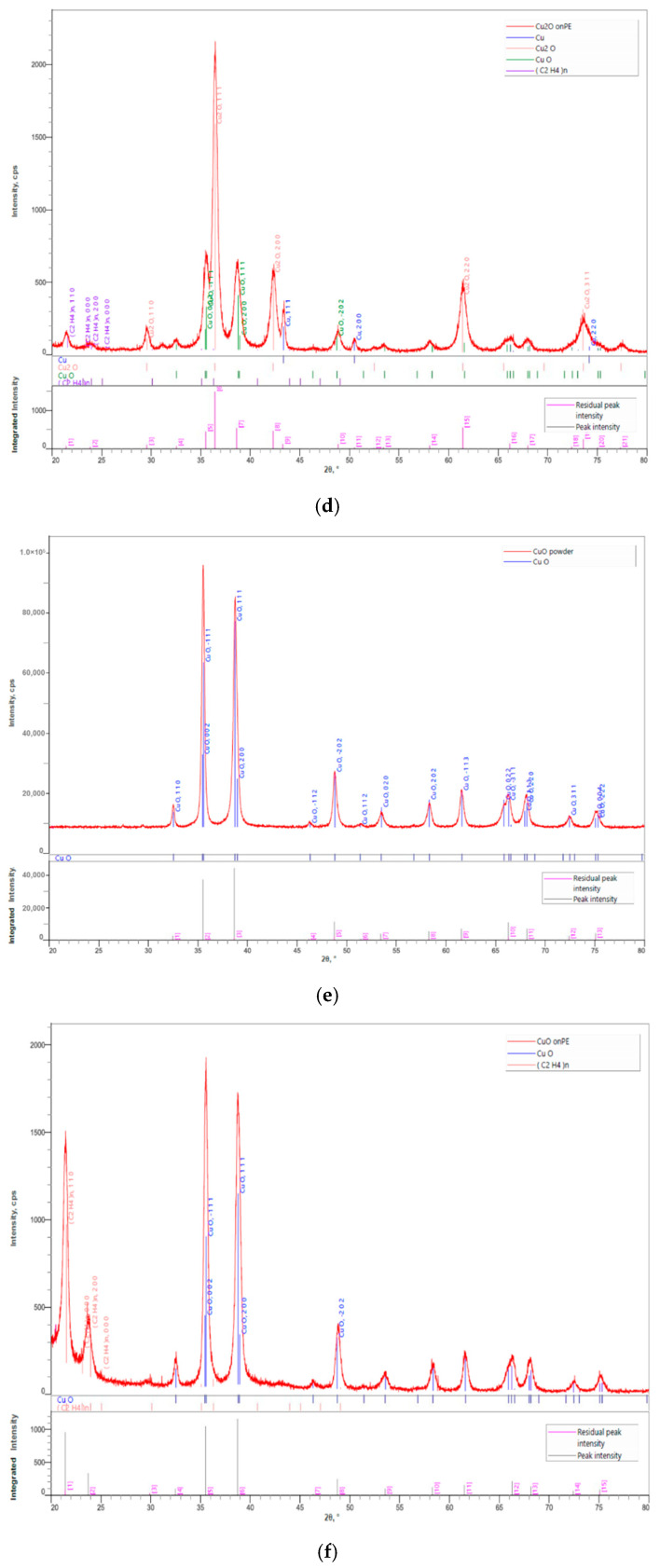
X-ray Diffraction (XRD) analysis of (**a**) CuNPs; (**b**) LLDPE/CuNPs; (**c**) Cu_2_ONPs; (**d**) LLDPE/Cu_2_ONPs; (**e**) CuONPs; (**f**) LLDPE/CuONPs.

XRD-analysis was performed in order to determine whether NPs undergo changes in their oxidation state during the course of immobilization performed by thermal adhesion. The obtained patterns are exhibited in Figure 3. Reference intensity ratios (RIR) were obtained from the semi-quantitative phase analysis of samples, whereas peaks of the LLDPE phase were not taken into account in calculations. RIR enabled evaluation of the sample composition (Table 1).

The main fraction in the case of powdery and immobilized CuNPs was Cu^0^ (Table 1, Figure 3a,b). However, Cu^+1^ was also detected (13% in powder and 26% in immobilized NPs). Thermal treatment probably caused partial oxidation of CuNPs to Cu^1+^. The weight fraction of the Cu^+1^ in the powdery and immobilized Cu_2_O was 53 and 54%, respectively (Table 1, Figure 3c,d). This result indicates that there was no significant change in the NPs’ state during the immobilization. Furthermore, no changes were observed in the composition of CuONPs before and after the immobilization (Table 1, Figure 3e,f).

### 3.2. Leaching of Copper from Immobilized NPs 

The presence of copper ions in aqueous solutions may be very problematic and may have an adverse effect on public health [28]. Drinking water containing copper in excessive concentrations can cause gastrointestinal disorders and lead to liver poisoning. According to the WHO, the permissible copper concentration in drinking water is up to 2 ppm only [29]. 

**Figure 4:**
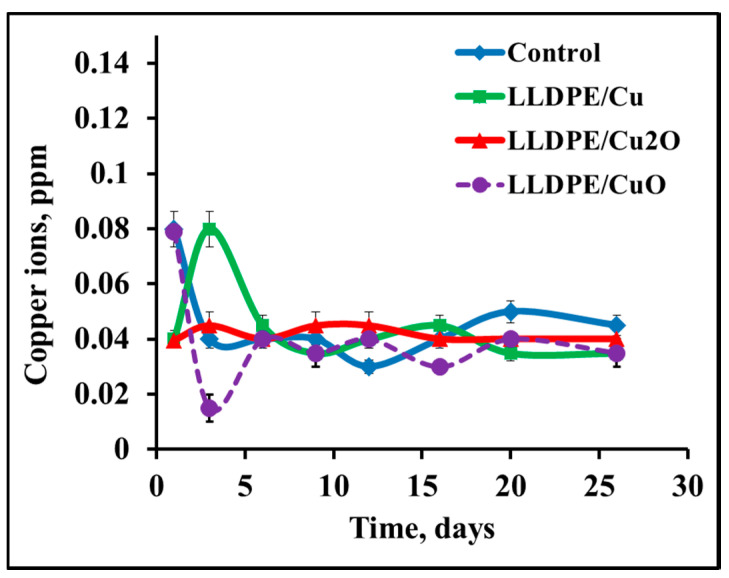
Leaching of copper ions into saline from composites of LLDPE and NPs of copper and copper oxides. Control—saline solution.

Due to this limitation, it was important to monitor leaching of copper ions into a saline solution from the prepared nanocomposites in the absence and presence of bacterial cells. Figure 4 presents results of testing the copper ion concentration during batch incubation of immobilized NPs in a saline solution for one month. No copper leakage into the saline solution was detected from any tested composite. In all cases, the copper concentration did not exceed 0.08 ppm, i.e., it remained below the permitted level and did not differ from the concentration in the control saline solution. 

**Figure 5:**
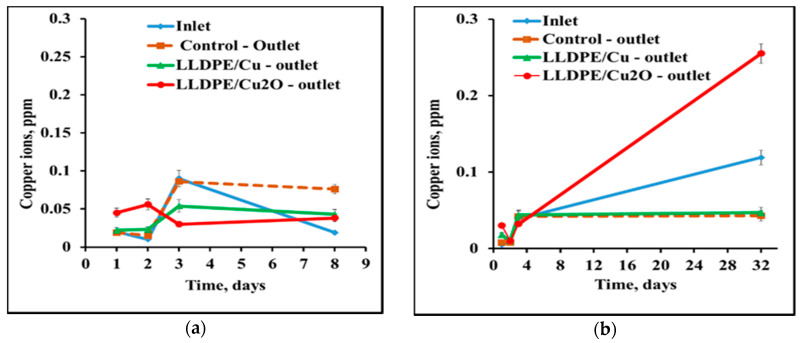
Leaching of copper ions from composites of LLDPE and NPs of copper and copper oxide into bacterial suspensions of (**a**) *S. aureus* and (**b**) *E. coli* in saline in a continuous experiment. Copper concentration was measured at the inlet to the system and at the outlets from the columns with composites LLDPE/CuNPs and LLDPE/Cu_2_ONPs, and from a control column not containing composites.

Leaching of copper ions in the presence of bacterial cells in saline solution was studied in a continuous regime. The composite rolls were placed into glass columns through which suspensions of bacterial cells in saline were transferred for several days. Presence of S. aureus cells in the saline did not affect the leaching of copper, and the results of the continuous experiment did not differ from the batch one with saline only (Figure 5a). In all cases, the copper concentration did not exceed 0.1 ppm. Presence of E. coli cells in the saline caused a slight release of copper ions from immobilized Cu_2_ONPs and the copper concentration reached 0.25 ppm on day 32. However, this concentration was still much lower than the upper permitted limit (Figure 5b).

### 3.3. Antimicrobial Activity of Free and Immobilized NPs of Copper and Its Oxides 

**Figure 6:**
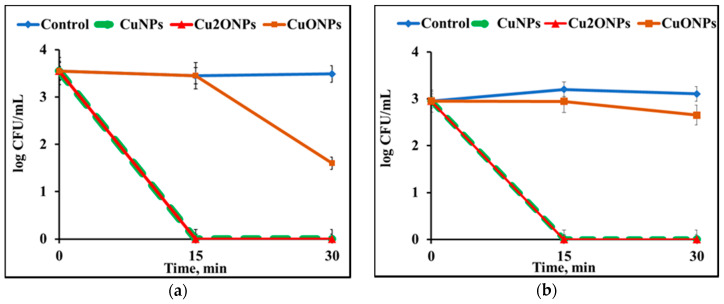
Activity of NPs of free copper and its oxides against (**a**) *S. aureus* and (**b**) *E. coli* cells at 25 °C. Control—untreated bacterial cells.

First, the free NPs were tested for antimicrobial activity in batch regime experiments against *S. aureus* and *E. coli*. It can be seen that free CuNPs and Cu_2_ONPs eradicated both *S. aureus* (Figure 6a) and *E. coli* (Figure 6b) after 15 min. CuONPs decreased the concentration of *S. aureus* cells by only 2 log_10_ after 30 min, and the concentration of *E. coli* cells was not reduced significantly even after 30 min. The difference in the antibacterial activity of LLDPE/CuONPs and LLDPE/Cu_2_ONPs may be associated with different oxidation states of copper in these composites. Meghana et al. assumed that enhanced antibacterial activity of Cu_2_O can be explained by its rapid binding to cell proteins, causing direct damage to the enzyme fumarase A, when this process takes place much faster than in the case of CuO [26]. 

**Figure 7:**
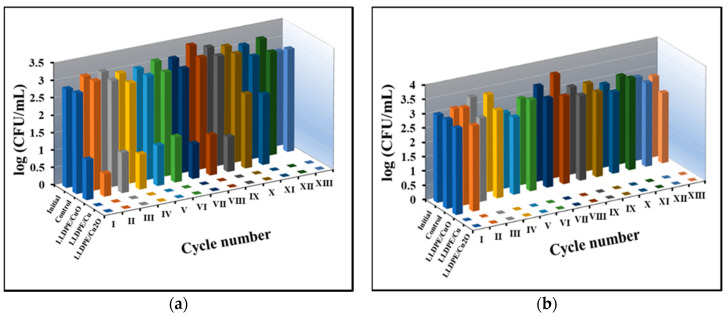
Activity of the NPs-LLDPE composites obtained by thermal adhesion against (**a**) *S. aureus* and (**b**) *E. coli* cells. Roman figures show the number of re-use cycles. Initial—*S. aureus* (**a**) and *E. coli* (**b**) cells before incubation, Control—*S. aureus* (**a**) and *E. coli* (**b**) cells after 30 min incubation; LLDPE/Cu_2_O—*S. aureus* (**a**) and *E. coli* (**b**) cells after 30 min incubation with the Cu_2_ONPs-LLDPE composite; LLDPE/Cu—*S. aureus* (**a**) and *E. coli* (**b**) cells after 30 min incubation with the CuNPs-LLDPE composite; LLDPE/CuO—*S. aureus* (**a**) and *E. coli* (**b**) cells after 30 min incubation with the CuONPs-LLDPE composite.

The results of the experiments with free NPs correlate well with the antibacterial activity of immobilized NPs tested in a batch regime (Figure 7). The latter experiments were carried out in order to study the re-usability of immobilized NPs, which was impossible in the case of free NPs. 

The composites were placed into suspensions of bacterial cells at known concentrations and incubated for half an hour, after which the bacterial concentration was measured. For testing re-usability, the composites were transferred into fresh suspensions of bacterial cells. It can be seen that immobilized CuONPs caused a decrease of approximately 2 log_10_ in the concentration of S. aureus cells during 9 cycles, and 1 log_10_ in cycles 10–11 of re-using the sample (Figure 6a), whereas it was inactive against E. coli (Figure 7b). The samples of immobilized CuNPs and Cu_2_ONPs were active against both bacteria and totally eradicated the S. aureus (Figure 7a) and E. coli (Figure 7b) cells for at least 13 cycles of re-use. 

The observed higher sensitivity of *S. aureus* to free and immobilized CuONPs compared to *E. coli* cells may be due to the different mechanisms of NPs action on these bacteria. Lv et al. reported that CuONPs doped with 5% Mg, 3% Zn, and 5% Ce by a hydrothermal method was more effective against *S. aureus* than against *E. coli* [30]. Furthermore, Ma et al. showed that copper-doped zinc oxide prepared by a sol-gel technique was less active against *E. coli* than against *S. aureus* because *E. coli* cells have a membrane with a bilayer structure, which provides resistance to antibacterial agents [31].

**Figure 8:**
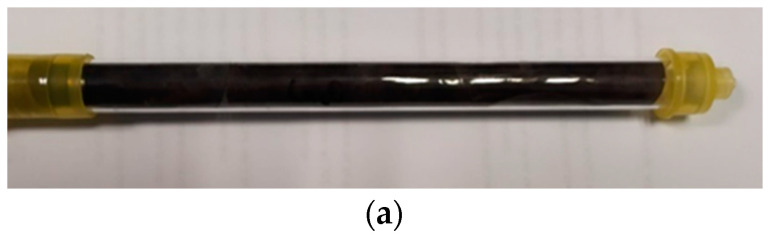

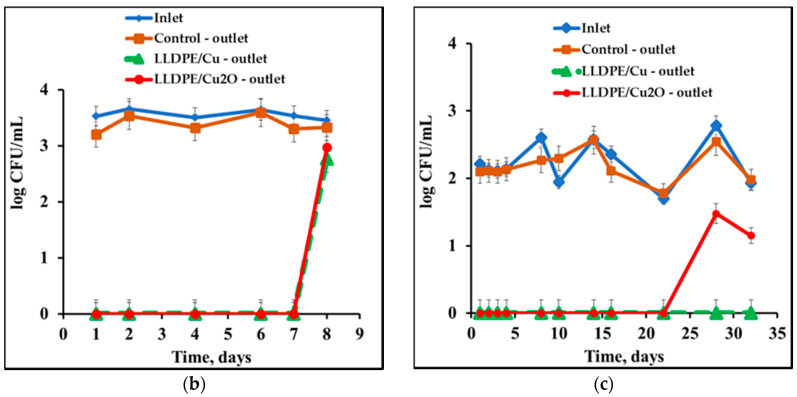
Antibacterial effect of CuNPs and Cu_2_ONPs immobilized onto LLDPE rolled into a column (**a**) against *S. aureus* (**b**) and *E. coli* (**c**) cells in a continuous regime. Cell concentration was measured at the inlet to the system and at the outlets of the columns with LLDPE/CuNPs and LLDPE/Cu_2_ONPs composites, and from a control column.

In the next stage of our study, immobilized CuNPs and Cu_2_ONPs which showed the highest activity against both bacteria in a batch mode were tested in a continuous regime against the same bacteria. For this purpose, rolled samples of immobilized NPs were placed into columns (Figure 8a), where another empty column served as a control of bacterial suspension. The columns were fed from the same source of bacterial cell suspensions of *S. aureus* or *E. coli*. Bacterial concentration was tested at the inlet and outlet of the columns. The results of these experiments are presented in Figure 8b,c. 

No live *S. aureus* bacteria were found at the column’s outlet, whereas the bacterial concentration at the outlet of the control column was the same as at the inlet (Figure 7b). Both CuNPs- and Cu_2_ONPs-based composites were active against *S. aureus* during seven days of the experiment. On day 8 they lost their antibacterial properties and the bacterial concentration at the outlet became close to that at the inlet. These composites also eradicated *E. coli* cells very effectively. Immobilized CuNPs retained their antibacterial activity for at least 32 days, and Cu_2_ONPs retained it for 22 days (Figure 8c). Immobilized CuNPs and Cu_2_ONPs were active against *E. coli* cells for a longer time than against *S. aureus* cells, probably due to slower oxidation of Cu and Cu_2_O to CuO in the *E. coli* suspension compared to the *S. aureus* suspension. 

We presume that the antibacterial activity of copper and copper oxide NPs is due to direct contact between NPs attached onto the polymeric surface and cells, and not because of the action of copper ions in solution on the cells, since no significant release of copper ions into the aqueous solution was observed in batch and continuous experiments. It should be noted that the exact mechanism of growth inhibition and eradication of bacteria by NPs of copper and its oxides is not totally clear. 

## 4. Conclusions

Nanocomposites based on linear low-density polyethylene with immobilized CuNPs, CuONPs and Cu_2_ONPs can be prepared by a simple thermal adhesion method. The obtained composites exhibit high antibacterial activity and show low release of copper ions into the aqueous phase, implying negligible health risk. In the batch experiments, Cu and Cu_2_O nanoparticles in suspensions as well as when immobilized onto LLDPE showed higher antibacterial activity than the CuO nanoparticles, probably due to the different oxidation states of the copper. In the continuous flow regime, the CuNPs and Cu_2_ONPs composites were active against *S. aureus* for seven days and against *E. coli* for 22 days. The high efficiency of the composites suggests that, after further optimizations and adjustments, they can be used for water disinfection in batch and continuous regimes.

## Figures and Tables

**Table 1:** Sample composition obtained by relative intensity ratio (RIR) measurements

Sample	Cu, wt%	Cu_2_O, wt%	CuO, wt%
Cu powder	87	13	ND ^1^
LLDPE/Cu	74	26	ND
Cu_2_O powder	4	53	43
LLDPE/Cu_2_O	4	54	42
CuO powder	ND	ND	100
LLDPE/CuO	ND	ND	100

^1^ ND—Not detected.

## Abbreviations

NPs	Nanoparticles
CuNPs	Copper nanoparticles
Cu_2_ONPs	Cuprous oxide nanoparticles
CuONPs	Copper oxide nanoparticles
LLDPE	Linear low-density polyethylene
LLDPE/CuNPs	Composite of LLDPE and CuNPs
LLDPE/Cu_2_ONPs	Composite of LLDPE and Cu_2_ONPs
LLDPE/CuONPs	Composite of LLDPE and CuONPs
CFU	Colony forming units
EDS	Energy-dispersive X-ray spectroscopy
SEM	Scanning electron microscope
RIR	Reference intensity ratios
ROS	Reactive oxygen species
WHO	World Health Organization
XRD	X-ray diffraction
